# Minimum dietary diversity and associated determinants among children aged 6–23 months in Pakistan

**DOI:** 10.1038/s41598-024-51705-4

**Published:** 2024-02-01

**Authors:** Ramesh Kumar, Tahir Mahmood, Nawal Naeem, Shahzad Ali Khan, Mubashir Hanif, Sathirakorn Pongpanich

**Affiliations:** 1 College of Public Health Sciences, Chulalongkorn University, Bangkok, 10330 Thailand; 2 Faculty of Business Administration, Sukkur IBA University, Sukkur, Pakistan; 3 Health Services Academy, Islamabad, Pakistan; 4 Department of Medical Genetics, University of Helsinki, Helsinki, Finland

**Keywords:** Health care, Medical research, Risk factors

## Abstract

Pakistan is facing a high prevalence of malnutrition and Minimum Dietary Diversity (MDD) is one of the core indicators that remain below the recommended level. This study assesses MDD and its associated factors among children aged 6 to 23 months in Pakistan. The study uses a cross-sectional study using the dataset of the latest available Multiple Indicators Cluster Survey (MICS) for all provinces of Pakistan. Multistage sampling is used to select 18,699 children aged 6 to 23 months. The empirical method is the Logistic Regression Analysis and Chi-Square Test. The dataset is freely and publicly available with all identifier information removed, and no ethics approvals are required. About one-fifth (20%) of infants and young children aged 6 to 23 months had met MDD, this number varies from 17 to 29%, highest in Baluchistan and lowest in Punjab province of Pakistan. The age group (18–23) indicates a 2.45 times greater chance of having MDD. Age (< 0.001), diarrhea (0.01), prenatal care (0.06), mother’s education (< 0.001), computer access (< 0.001), wealth quantile (< 0.001), and residence (< 0.001) were significantly associated with meeting MDD. However, gender (0.6) and mother’s age (0.4) both were statistically insignificant in meeting MDD. Regarding mothers’ education, compared to no education, the chance of MDD is 1.45 times greater for highly educated mothers in the Punjab province. Dietary diversity among children aged 6 to 23 months in Pakistan is low. It is recommended that mothers should be aware and encouraged to use dietary diverse food for infants and younger children.

## Introduction

Malnutrition is considered a major public health problem among children less than five years of age. Nearly half of the deaths are reported due to undernutrition, especially in developing countries globally^1,2^. The World Health Organization (WHO) has recommended; breastfeeding and complementary feeding as a core of Infant Young Child Feeding (IYCF) practices among children aged 6–23 months^3^. Moreover, Sustainable Development Goal (SDG) 2 is highly focused on the importance of diet and zero hunger^4^. The initial 2 years of childhood are considered an alarming time that needs special focus on foods for normal growth and development^3^. Hence, an appropriate diet can positively improve the health of children below two years of age and help in the substantial reduction of morbidity and mortality of children^2^.

Globally, very few children are eating diversified food as per the WHO criteria. Many countries reported less than one-quarter of their children aged 6–23 months using dietary diversity^5^. Parents from low and middle-income countries are struggling to follow Minimum Dietary Diversity (MDD) as per the recommendation due to scarce resources^6^. Dietary diversity is one of the identified components and major factors responsible for the dietary pattern among the children who consumed several food items during the last day^2^. Dietary practices of children under two years of age are strongly correlated with nutritional status and child survival. MDD is defined as the eating of five or more food groups from the eight food groups for required daily energy for children aged 6–23 months. These eight food groups were: breast milk; grains, roots, and tubers; legumes and nuts; dairy products (milk, yogurt, cheese); flesh foods (meat, fish, poultry, and liver/organ meats); eggs; vitamin-A rich fruits and vegetables; other fruits and vegetables^7,8^. Lacking dietary diversity in the diet is responsible for developing undernutrition including stunting, underweight, and wasting in children^9^. Hence, this number is quite high in low- and middle-income countries where there is limited access to dietary diversity^2,10^. One out of four children aged 6–23 months are taking the minimum dietary diversity as per the WHO guidelines in low and middle-income countries^11^. The nutritional status of children could be improved by eating diversified food as per WHO recommendations^12^. Poor dietary diversity is associated with stunting and underweight among children under five years of age. Research proved that children who do not follow the minimum dietary diversity in their regular food habits are at high risk of being stunted, underweight, anemic, easily getting infections, and severe illnesses^11^.

Though the Pakistani government has worked for the betterment of children’s dietary diversity, it always remained a big challenge and reported that the lowest adequate dietary diversity includes; exclusive breastfeeding (48.4%), complementary feeding practices, such as MDD (14.2%), Minimum Meal Frequency (MMF) (18.2%) and Minimum Acceptable Diet (MAD) (3.6%)^13,14^. Studies were conducted to assess the associated determinants of dietary diversity among children aged 6–23 months^2,10^. However, none of the studies conducted in Pakistan is available based on national representative data. Hence, we investigated the community and individual-level determinants of minimum dietary diversity among children aged 6–23 months in all four provinces of Pakistan by using national representative survey data with advanced statistical analysis of Multi Indicator Cluster Surveys (MICS).

## Methodology

### Study design

This is a cross-sectional study design with secondary data analysis utilizing the latest available data of MICS of four provinces of Pakistan. Available datasets from all the provinces include; Multi Indicator Cluster Survey-Khyber Pakhtunkhwa (MICS-KP-2019)^15^, Multi Indicator Cluster Survey-Baluchistan (MICS-Baluchistan-2019–2020)^16^, Multi Indicator Cluster Survey- Sindh (MICS-Sindh-2018–2019)^17^, and Multi Indicator Cluster Survey-Punjab (MICS-Punjab-2017–2018)^18^, collected by the Bureau of Statistics, in collaboration with United Nations International Children's Emergency Fund (UNICEF), as part of the Global Multi Indicator Cluster Survey (MICS) Programme. UNICEF with government funding and financial support of UNICEF, provided technical support. A single file for the whole of Pakistan has been generated which equates to 18,699 children aged 6 to 23 months for four provinces using multistage samplings. The composition of four provinces; Khyber Pakhtunkhwa (KP), Baluchistan, Sindh, and Punjab are 4616, 2696, 3068, and 8219, respectively. All methods were carried out in accordance with relevant guidelines and regulations.

### Statistical analysis

This study utilizes the Chi-Square test and Logistic Regression Analysis to discuss the prevalence of MDD and determinants of MDD in Pakistan as well as four provinces of Pakistan. The Chi-Square statistic is a pivotal test to gauge Tests of Independence using a cross-tabulation. The cross-tabulation captures the distributions of two categorical variables. The Test of Independence evaluates whether a relationship exists between two variables^19^. A logistic regression predicts future outcomes, assesses the statistical significance of covariate variables, and involves the prediction of a binary outcome variable. The covariates can be continuous or binary, just as in regression analysis, but Ordinary Least Squares regression (OLS) is not appropriate if the outcome variable is binary^19^.

### Covariates

The demographic and socio-economic determinants of the minimum dietary diversity used in the analysis are; child age in months, classified into three age categories; sex of the child; the child had diarrhea in the last 2 weeks; received prenatal care; mother’s education, mother’s age; ever used a computer or a tablet; household have electricity; wealth index quintile and residence of the households and four provinces.

### Ethics approval and consent to participate

This study used MICS survey data. The Bureau of Statistics Institutional Review Board (IRB) approved the data collection process, which involved obtaining informed consent from respondents. This study is based on an analysis of cross-sectional data available freely and publicly with all identifier information removed, no ethics approvals were required.

## Results

This study utilizes the latest available MICS datasets for four provinces of Pakistan. The results section starts with the statistical description of the outcome variable, that is MDD index and covariates that is individual and household characteristics. MDD index is defined as receiving foods from at least 5 out of 8 food groups: (1) breast milk, (2) grains, roots, and tubers, (3) legumes and nuts, (4) dairy products (milk, infant formula, yogurt, cheese) (5) flesh foods (meat, fish, poultry and liver/organ meats), (6) eggs, (7) vitamin-A rich fruits and vegetables, and (8) other fruits and vegetables, consumed in the 24 h preceding the survey. The generated MDD index using the above eight food items takes on two values; 0 and 1. Zero stand for a child aged 6–23 months receiving less than five food items, while 1 represents a child aged 6–23 months receiving five or more than five food items. Thus, a child receiving at least five food items qualifies for fulfilling minimum dietary diversity intake. The distribution of these eight food items for the whole dataset is given in Table 1.

**Table 1:** The distribution of different food items used in the construction of the MDD Index.

Food items	Pakistan	KP	Baluchistan	Sindh	Punjab
Breast Milk	0.720	0.770	0.793	0.806	0.635
Grains, roots, and tubers	0.686	0.670	0.556	0.684	0.739
Legumes and nuts	0.104	0.113	0.171	0.143	0.061
Dairy products	0.920	0.938	0.883	0.891	0.933
Flesh foods	0.134	0.102	0.258	0.151	0.104
Eggs	0.270	0.215	0.292	0.242	0.305
Vitamin-A-rich fruits and vegetables	0.319	0.372	0.375	0.167	0.328
Other fruits and vegetables	0.174	0.185	0.266	0.237	0.113

Almost 72% of the mothers, breastfed their children aged 6–23 months in Pakistan. The percentage of breastfeeding was highest for Sindh province (80%), while lowest for Punjab province, which is almost 63%. Among the eight food items, the highest proportion of food taken by children aged 6–23 months is the dairy product, which is 92% for Pakistan. The similar highest percentage is also for the provinces. The percentage of flesh food intake is the lowest, in that the average of the four provinces is about 13%. However, flesh food intake is almost double for Baluchistan province compared to the average score and other provinces (Table 1).

The MDD index for the whole sample is 20%, while substantial variation exists among four provinces; that is highest in Baluchistan (29%), KP (21%), Sindh (20%), and lowest in Punjab (17%). For gender, there is no significant variation across the provinces. Prenatal care facilities are much better in Punjab compared to KP and Baluchistan. Similarly, mother education is the lowest in Baluchistan; that is 85% of mothers are uneducated in Baluchistan. The percentages of households having no access to electricity are 13% and 16% in Baluchistan and Sindh provinces, respectively. The wealth quintile from poorest to richest shows a constant decline in the percentage of children aged 6–23 months in specific wealth groups for the whole sample as well as for provinces. Whereas, almost 75% of the dataset is collected from rural areas for the whole sample, while province-wise data show enough variation regarding urban–rural distribution. Table 2 presents the results of the Chi-Square test that demonstrates the relationship and interdependence between covariates and the MDD index using cross-tabulation. For instance, the second column of Table 3 shows Chi-Square test results for the whole sample (Pakistan), further classified the individuals and households characteristics by the number of children that fall short of the recommended MDD represented by *No* (80%) and those children who fulfill the recommended MDD represented by *Yes* (20%). For example, meeting MDD intake rises significantly with the increasing age group of children such as for age group 6–11 (12%), age group 12–17 (23%) and age group 18–23 (24%). Similarly, meeting MDD intake increases in conjunction with increased prenatal care, significant at 5% for Punjab province (17%). Moreover, the analysis establishes no significant interdependence between various mother age groups, child gender, child with diarrhea, and MDD intake. Importantly, there is a significant relationship between mother schooling and MDD usage for Sindh, Baluchistan, and Punjab, except KP province. In terms of computer accessibility and MDD utilization, the Punjab region outperforms other provinces. Similarly, for all provinces, there is an increasing positive relationship between various wealth index categories and MDD intake. For instance, in case of Sindh province, for the poorest wealth quantile it is only 14%, while it is almost 30% for the richest wealth quintile.

**Table 2:** Chi-Square test results showing the relationship between the MDD Index and individual and household characteristics.

Variables	Pakistan			KP				Baluchistan			Sindh			Punjab		
	MDD index (types of food intake > 4)															
	Total N	No N (%)	Yes N (%)	Total		No N (%)	Yes N (%)	Total N	No N (%)	Yes N (%)	Total N	No N (%)	Yes N (%)	Total N	No N (%)	Yes N (%)
	18,699	14,957 (80)	3742 (20)	4716		3735 (79)	981 (21)	2696	1909 (71)	787 (29)	3068	2460 (80)	608 (20)	8219	6853 (83)	1366 (17)
Age group	< 0.001			< 0.001				< 0.001			< 0.001			< 0.001		
6–11 months	5976	5263 (88.1)	713 (11.9)	1618	1417 (87.6)		201 (12.4)	768	604 (78.7)	164 (21.4)	1040	920 (88.5)	120 (11.5)	2550	2322 (91.1)	228 (8.9)
12–17 months	7429	5690 (76.6)	1739 (23.4)	1823	1379 (75.6)		444 (24.4)	1270	863 (67.9)	407(32.1)	1165	879 (75.5)	286 (24.6)	3171	2569 (81.0)	602 (18.9)
18–23 months	5294	4004 (75.6)	1290 (24.4)	26.35	939 (73.7)		336 (26.4)	658	442 (67.2)	216 (32.8)	863	661(76.6)	202 (23.4)	2498	1962 (78.5)	536 (21.5)
Gender	0.663			0.178				0.088			0.200			0.055		
Female	9180	7331 (79.9)	1849 (20.1)	2316	1853 (80.0)		463 (19.9)	1357	981 (72.3)	376 (27.7)	1508	1195 (79.2)	313 (20.8)	3999	3302 (82.6)	697 (17.4)
Male	9519	7626 (80.1)	1893 (19.9)	2400	1882 (78.4)		518 (21.6)	1339	928 (69.3)	411 (30.7)	1560	1265 (81.1)	295 (18.9)	4220	3551 (84.2)	669 (15.9)
Diarrhea	0.019			0.002				< 0.001			0.134			0.456		
No	14,249	11,452 (80.4)	2797 (19.6)	2883	2325 (80.7)		558 (19.4)	2234	1617 (72.4)	617 (27.6)	2562	2042 (79.7)	520 (20.3)	6570	5468 (83.2)	1102 (16.8)
Yes	4450	3505 (78.8)	945 (21.2)	1833	1410 (76.9)		423 (23.1)	462 |	292 (63.2)	170 (36.8)	506	418 (82.6)	88 (17.4)	1649	1385 (83.9)	264 (16.0)
Prenatal care	0.061			0.536				0.776			0.170			0.026		
No	12,078	9612 (79.6)	2466 (20.4)	3989	3153 (79.0)		836 (20.9)	2111	1492 (70.7)	619 (29.3)	2154	1741 (80.8)	413 (19.2)	3824	3226 (84.4)	598 (15.6)
Yes	6621	5345 (80.7)	1276 (19.3)	727	582 (80.1)		145 (19.9)	585	417 (71.3)	168 (28.7)	914	719 (78.7)	195 (21.3)	4395	3627 (82.5)	768 (17.5)
Mother’s age	0.494			0.606				0.598			0.946			0.605		
15–19 years	2586	2063 (79.8)	523 (20.2)	782	622 (79.5)		160 (20.5)	384	269 (70.1)	115 (29.9)	500	406 (81.2)	94 (18.8)	920	766 (83.3)	154 (16.7)
20–24 years	3530	2819 (79.9)	711 (20.1)	860	667 (77.6)		193 (22.4)	492	349 (70.9)	143 (29.1)	543	429 (79.0)	114 (20.9)	1635	1374 (84.0)	261 (15.9)
25–29 years	4324	3461 (80.0)	863 (19.9)	889	701 (78.9)		188 (21.2)	588	418 (71.1)	170 (28.9)	662	527 (79.6)	135 (20.4)	2185	1815 (83.1)	370 (16.9)
30–34 years	3421	2744 (80.2)	677 (19.8)	765	602 (78.7)		163 (21.3)	458	339 (74.0)	119 (25.9)	494	400 (80.9)	94 (19.0)	1704	1403 (82.3)	301 (17.7)
35–39 years	2477	2001 (80.8)	476 (19.2)	670	540 (80.6)		130 (19.4)	366	256 (69.9)	110 (30.1)	402	323 (80.4)	79 (19.7)	1039	882 (84.9)	157 (15.1)
40–44 years	1304	1015 (77.8)	289 (22.2)	405	319 (78.8)		86 (21.2)	224	149 (66.5)	75 (33.5)	253	200 (79.1)	53 (20.9)	422	347 (82.2)	75 (17.8)
45–49 years		854 | (80.8)	203 (19.2)	345	284 (82.3)		61 (17.7)	184	129 (70.1)	55 (29.9)	214	175 (81.8)	39 (18.2)	314	266 (84.7)	48 (15.3)
Mother’s education	< 0.001			0.518				0.008			< 0.001			< 0.001		
None	10,815	8710 (80.5)	2105 (19.5)	3089	2459 (79.6)		630 (20.4)	2297	1600 (69.7)	697 (30.3)	1994	1635 (82.0)	359 (18.0)	3435	3016 (87.8)	419 (12.2)
Primary	2699	2231 (82.7)	468 (17.3)	540	432 (80.0)		108 (20.0)	134	98 (73.1)	36 (26.9)	368	309 (83.9)	59 (16.0)	1657	1392 (84.0)	265 (15.9)
Middle	1365	1082 (79.2)	283 (20.7)	304	240 (78.9)		64 (21.1)	67	50 (74.6)	17 (25.4)	147	110 (74.8)	37 (25.2)	847	682 (80.5)	165 (19.5)
Secondary	1911	1518 (79.4)	393 (20.6)	373	283 (75.9)		90 (24.1)	112	94 (83.9)	18 (16.1)	251	198 (78.9)	53 (21.1)	1175	943 (80.3)	232 (19.7)
Higher	1909	1416 (74.2)	493 (25.9)	410	321 (78.29)		89 (21.7)	86	67 (77.9)	19 (22.1)	308	208 (67.5)	100 (32.5)	1105	820 (74.2)	285 (25.8)
Computer access	< 0.001			0.462				0.287			0.451			< 0.001		
No	17338	13,939 (80.4)	3399 (19.6)	4413	3490 (79.1)		923 (20.92)	2551	1812 (71.0)	739 (28.9)	2886	2318 (80.3)	568 (19.7)	7488	6319 (84.4)	1169 (15.6)
Yes	1361	1018 (74.8)	343 (25.2)	303	245 (80.9)		58 (19.1)	145 |	97 (66.9)	48 (33.1)	182	142 (78.0)	40 (21.9)	731	534 (73.1)	197 (26.9)
Electricity access	0.032			0.721				0.626			0.003			< 0.001		
Yes	17,358	13,854 (79.8)	3504 (20.2)	4593	3636 (79.2)		957 (20.8)	2336	1658 (70.9)	678 (29.0)	2563	2031 (79.2)	532 (20.8)	7866	6529 (83.0)	1337 (17.0)
No	1341	1103 (82.3)	238 (17.8)	123	99 (80.5)		24 (19.5)	360	251 (69.7)	109 (30.3)	505	429 (84.9)	76 (15.1)	353	324 (91.8)	29 (8.2)
Wealth quintile	< 0.001			< 0.001				< 0.001			0.000			0.000		
Poorest	4591	3734 (81.3)	857 (18.7)	1140	859 (75.4)		281 (24.7)	824	540 (65.5)	284 (34.5)	711	608 (85.5)	103 (14.5)	1916	1727 (90.1)	189 (9.9)
Poorer	4172	3397 (81.4)	775 (18.6)	968	770 (79.5)		198 (20.5)	631	440 (69.7)	191 (30.3)	816	673 (82.5)	143 (17.5)	1757	1514 (86.2)	243 (13.8)
Middle	3794	3057 (80.6)	737 19.4	909	766 (84.3)		143 (15.7)	496	330 (66.5)	166 (33.5)	640	511 (79.8)	129 (20.2)	1749	1450 (82.9)	299 (17.1)
Richer	3361	2668 (79.4)	693 (20.6)	929	736 (79.2)		193 (20.8)	384	307 (79.9)	77 (20.0)	502	388 (77.3)	114 (22.7)	1546	1237 (80.0)	309 (19.9)
Richest	2781	2101 (75.6)	680 (24.5)	770	604 (78.4)		166 (21.6)	361	292 (80.9)	69 (19.1)	399	280 (70.2)	119 (29.8)	1251	925 (73.9)	326 (26.1)
Residence	< 0.001			0.442				0.181			< 0.001			< 0.001		
Rural	14,044	11,324 (80.6)	2720 (19.4)	4139	3271 (79.0)		868 (20.9)	2096	1471 (70.2)	625 (29.8)	1713	1432 (83.6)	281 (16.4)	6096	5150 (84.5)	946 (15.5)
Urban	4655	3633 (78.1)	1022 (21.9)	577	464 (80.4)		113 (19.6)	600	438 (73.0)	162 (27.0)	1355	1028 (75.9)	327 (24.1)	2123	1703 (80.2)	420 (19.8)

**Table 3:** Logistic regression analysis results for whole sample and four provinces.

Variables	Pakistan	KP	Baluchistan	Sindh	Punjab
Reference category (6–11 months)					
12–17 months	2.233*** [2.029,2.457]	2.268*** (1.887–2.725)	1.778*** (1.436–2.203)	2.435*** (1.925–3.081)	2.399*** (2.035–2.827)
18–23 months	2.455*** [2.219,2.717]	2.574*** [2.118,3.126]	1.796*** [1.410,2.287]	2.350*** [1.829,3.018]	2.867*** [2.422,3.393]
Reference category (Female)					
Male	0.983 [0.914,1.057]	1.107 [0.959,1.278]	1.148 [0.968,1.361]	0.909 [0.757,1.090]	0.890 [0.790,1.002]
Diarrhea	Reference category (No)				
Yes	1.137*** [1.043,1.241]	1.273** [1.101,1.473]	1.491** [1.202,1.851]	0.931 [0.720,1.203]	1.042 [0.896,1.213]
Prenatal care	Reference category (No)				
Yes	1.044 [0.957,1.138]	0.891 [0.725,1.097]	1.081 [0.871,1.342]	1.074 [0.873,1.321]	1.084 [0.950,1.236]
Mother’s age	Reference category (15–19 years)				
20–24 years	1.000 [0.877,1.140]	1.130 [0.886,1.440]	0.873 [0.644,1.182]	1.192 [0.867,1.638]	0.890 [0.707,1.121]
25–29 years	0.989 [0.870,1.125]	1.089 [0.851,1.392]	0.880 [0.655,1.182]	1.059 [0.778,1.441]	0.910 [0.728,1.139]
30–34 years	0.988 [0.864,1.130]	1.074 [0.833,1.384]	0.746 [0.546,1.021]	0.969 [0.696,1.350]	0.990 [0.786,1.248]
35–39 years	0.929 [0.805,1.072]	0.928 [0.712,1.208]	0.928 [0.671,1.282]	1.044 [0.741,1.471]	0.845 [0.654,1.092]
40–44 years	1.102 [0.933,1.301]	1.047 [0.775,1.414]	1.052 [0.733,1.511]	1.172 [0.796,1.726]	1.154 [0.843,1.579]
45–49 years	0.920 [0.765,1.106]	0.817 [0.586,1.140]	0.921 [0.621,1.366]	1.028 [0.673,1.570]	0.895 [0.624,1.284]
Mother’s education	Reference category (No education)				
Primary	1.028 [0.912,1.158]	1.053 [0.828,1.338]	0.998 [0.660,1.510]	0.734 [0.534,1.009]	1.129 [0.945,1.350]
Middle	1.269*** [1.090,1.477]	1.168 [0.859,1.588]	1.053 [0.591,1.878]	1.134 [0.745,1.726]	1.254* [1.006,1.563]
Secondary	1.225 [1.066,1.408]	1.397* [1.060,1.843]	0.612 [0.356,1.052]	0.881 [0.605,1.283]	1.168 [0.947,1.441]
Higher	1.604 [1.394,1.846]	1.232 [0.927,1.638]	0.867 [0.500,1.504]	1.428* [1.018,2.001]	1.456*** [1.168,1.816]
Computer access	Reference category (No)				
Yes	1.283*** [1.122,1.467]	0.867 [0.641,1.172]	1.294 [0.894,1.872]	0.952 [0.652,1.391]	1.478*** [1.220,1.790]
Electricity access	Reference category (Yes)				
No	0.835** [0.706,0.988]	0.714 [0.445,1.144]	0.771 [0.580,1.025]	1.055 [0.726,1.533]	0.847 [0.558,1.284]
Wealth quintile	Reference category (Poorest)				
Poorer	0.938 [0.834–1.055]	0.727** [0.586,0.901]	0.712** [0.557,0.911]	1.287 [0.905,1.830]	1.399** [1.125,1.741]
Middle	0.956 [0.844,1.084]	0.518*** [0.409,0.657]	0.797 [0.609,1.044]	1.433 [0.974,2.109]	1.678*** [1.339,2.103]
Richer	0.953 [0.831,1.093]	0.704** [0.558,0.889]	0.361*** [0.256,0.508]	1.425 [0.920,2.207]	1.976*** [1.547,2.524]
Richest	1.040 [0.893,1.219]	0.709* [0.538,0.933]	0.341*** [0.234,0.498]	1.921** [1.192,3.095]	2.498*** [1.899,3.285]
Residence	Reference category (Rural)				
Urban	1.068 [0.969,1.178]	0.933 [0.734,1.187]	1.450** [1.130,1.861]	1.265* [1.005,1.592]	0.884 [0.760,1.028]
Provinces	Reference category (KP)				
Baluchistan	1.668*** [1.486- 1.872]				
Sindh	0.957 [0.844- 1.084]				
Punjab	0.694*** [0.626,0.769]				
Constant	0.128*** [0.109, 0.151]	0.157*** [0.120,0.205]	0.361*** [0.260,0.501]	0.0843*** [0.0545,0.130]	0.0559*** [0.0423,0.0739]
n	18,699	4716	2696	3068	8219

Exponentiated coefficients; 95% confidence intervals in brackets.

*p < 0.05, **p < 0.01, ***p < 0.001.

Furthermore, there are no significant variations in household composition between urban and rural zones in the provinces of KP and Baluchistan. In contrast, there are significant differences between urban and rural regions in Punjab and Sindh provinces, implying that urban households rely heavily on MDD use. Similarly, for the Punjab and Sindh provinces, there is a significant positive relationship between electricity accessibility and meeting MDD. For instance, in the case of Punjab province, the MDD intake is only 8% with no access to electricity compared with 17% with access to electricity. Importantly, rather than the mother's education, the wealth of a household plays a greater part in MDD intake.

Table 3 presents the results of logistic regression analysis in the Odd Ratio (OR). For instance, when compared to the reference age group (6–11 months), the age group (18–23) has a 2.45 odds ratio, indicating a 2.45 times higher chance of meeting MDD. The same significant findings can be found in all provinces. The probability of meeting MDD for a child who has had diarrhea in the last 24 h is 1.27 and 1.49 times higher in KP and Baluchistan, respectively, and 1.13 times higher in the overall sample. In terms of education, highly educated mothers in the Punjab region have 1.45 times the chance of meeting MDD as illiterate mothers. Importantly, for the entire population, education increases the likelihood of meeting MDD. Whereas, the recommended MDD for Punjab is 1.47 times higher for those households having access to a computer. However, the mother’s age does not add any significant contributory factor in fulfilling the MDD intake. Moreover, the wealth of the household is a significant contributor to the likelihood of MDD for the whole sample as well as for four provinces. For instance, in the case of Punjab province, it is 2.49 times higher for the richest wealth quintile compared to the poorest wealth quintile. While it is 1.92 times higher for Sindh province for the same wealth quintile comparison.

## Discussion

Using nationally representative MICS data, this research investigated the MDD and its associated determinants of dietary diversity. We discovered that only 20% of children aged 6–23 months have sufficient minimum dietary diversity at the national level, with figures ranging from 17 to 29% in each of the four provinces. Furthermore, we discovered that the child's age, mother's education, computer access, prenatal care, residence, and family wealth index were all significantly related to the minimum dietary diversity of children. The prevalence of minimum dietary diversity was almost similar in this study to the National Nutrition Survey (NNS) report 2019^13^ and slightly lower than the Pakistan demographic health survey 2018^20^. Our findings were supported by other countries like Ethiopia, Nigeria, and Tanzania^2,21,22^.

This analysis showed that children aged 18–23 months presented a higher dietary diversity than infants aged 6–11 months. This finding is similar where the dietary diversity was high in the age group 18–23 months^2^. Another study also supports our findings and this number is further increased by the study results from Haramaya town, Ethiopia^23^. The possible explanation for this difference could be that the minimum dietary diversity changes with the child’s age after the breastfeeding stage. The study also shows that the dietary diversity of children changes as they grow^2,23^. For those mothers who had taken Antenatal Care (ANC) visits and got IYCF information, their children's food diversity was good as compared to mothers who did not. Other regional studies have also supported these findings^2,23,24^. Proper information is given during ANC visits that could improve their practices toward the dietary diversity of their children. Hence the study revealed that ANC information has a positive significance with MDD. This evidence supports our findings^2^. Hence, health education and counseling of mothers on nutrients and feeding is a significant activity in our country, this should be practiced in routine to achieve better food diversity in children. Children living in rural populations are highly exposed to MDD as compared to those who are living in urban areas in this study. This might be due to different factors like; poverty, non-availability of diverse food items, and affordability of families. MDD increases significantly with an increase in the age of the children. Previous studies have also confirmed this relationship^2,9^. A study from the same country also supports that poverty is a very important determinant of poor diet and education that results in stunting and wasting^25^. Importantly, a strong relationship exists among mother education different categories and MDD usage for all provinces except KP province. Punjab province outperforms other provinces regarding computer accessibility and MDD utilization. This is evident from the fact that Punjab is the richest province among others^13^. Similarly, among different wealth index categories, there exists a significant relationship with MDD intake for all provinces. Which is also confirmed by different studies^2,13^. The utilization of various kinds of foods has been associated with better nutritional outcomes among children^26^. In provinces like Sindh and Baluchistan, people from rural areas mostly migrate from one area to another or from rural to urban areas that may experience a food crisis^27^.

Mother’s education positively affects food insecurity, as compared to no education, the chance of meeting MDD is 1.45 times greater for highly educated mothers in Punjab province. Hence nutrition education plays an important role in a child’s dietary diversity in the community. Literature proved that poor food diversity is often seen in low-wealth groups and uneducated families groups^28^. Another research is also consistent with our findings and shows that MDD was correlated with the level of education. Those educated mothers who had at least a graduate level education were using diverse diets for their children as compared to those who had no schooling^26^. This might be due to their level of exposure and a better understanding of the importance of diversified food and its consumption for their children.

## Conclusion:

This analysis indicates that the consumption of minimum dietary diversity is lower than what is suggested at both the national and provincial levels. Furthermore, the age of the child, the mother's schooling, computer access, prenatal care, residence, and the household wealth index were all highly related to the minimum dietary diversity. As a result, more emphasis should be placed on raising community awareness of food diversity to overcome this problem.

## Data Availability

The datasets used are publicly available. https://microdata.worldbank.org/index.php/catalog/4181.
